# Comparison of saccharification and fermentation of steam exploded rice straw and rice husk

**DOI:** 10.1186/s13068-016-0599-6

**Published:** 2016-09-05

**Authors:** Ian P. Wood, Huong-Giang Cao, Long Tran, Nicola Cook, Peter Ryden, David R. Wilson, Graham K. Moates, Samuel R. A. Collins, Adam Elliston, Keith W. Waldron

**Affiliations:** 1 The Biorefinery Centre, Institute of Food Research, Norwich Research Park, Colney, Norwich, NR4 7UA UK; 2 Vietnam Academy of Agricultural Science, Hanoi, Vietnam; 3 The Earlham Institute, Norwich Research Park, Norwich, NR4 7UG UK

**Keywords:** Bioethanol, Biomass, Saccharification, Fermentation, Rice straw, Rice husk, Steam explosion, Pretreatment

## Abstract

**Background:**

Rice cultivation produces two waste streams, straw and husk, which could be exploited more effectively. Chemical pretreatment studies using rice residues have largely focussed on straw exploitation alone, and often at low substrate concentrations. Moreover, it is currently not known how rice husk, the more recalcitrant residue, responds to steam explosion without the addition of chemicals.

**Results:**

The aim of this study has been to systematically compare the effects of steam explosion severity on the enzymatic saccharification and simultaneous saccharification and fermentation of rice straw and husk produced from a variety widely grown in Vietnam (*Oryza sativa*, cv. KhangDan18). Rice straw and husk were steam exploded (180–230 °C for 10 min) into hot water and washed to remove fermentation inhibitors. In both cases, pretreatment at 210 °C and above removed most of the noncellulosic sugars. Prolonged saccharification at high cellulase doses showed that rice straw could be saccharified most effectively after steam explosion at 210 °C for 10 min. In contrast, rice husk required more severe pretreatment conditions (220 °C for 10 min), and achieved a much lower yield (75 %), even at optimal conditions. Rice husk also required a higher cellulase dose for optimal saccharification (10 instead of 6 FPU/g DM). Hemicellulase addition failed to improve saccharification. Small pilot scale saccharification at 20 % (w/v) substrate loading in a 10 L high torque bioreactor resulted in similarly high glucose yields for straw (reaching 9 % w/v), but much less for husk. Simultaneous saccharification and fermentation under optimal pretreatment and saccharification conditions showed similar trends, but the ethanol yield from the rice husk was less than 40 % of the theoretical yield.

**Conclusions:**

Despite having similar carbohydrate compositions, pretreated rice husk is much less amenable to saccharification than pretreated rice straw. This is likely to attenuate its use as a biorefinery feedstock unless improvements can be made either in the feedstock through breeding and/or modern biotechnology, or in the pretreatment through the employment of improved or alternative technologies. Physiological differences in the overall chemistry or structure may provide clues to the nature of lignocellulosic recalcitrance.

**Electronic supplementary material:**

The online version of this article (doi:10.1186/s13068-016-0599-6) contains supplementary material, which is available to authorized users.

## Background

Rice is the third most widely grown cereal crop in the world after maize and wheat. It is the staple food and a considerable source of income for many tropical nations. Very large quantities of agricultural lignocellulosic residues are generated from rice cultivation. Annually, paddy rice cultivation produces over 660 million tonnes of rice, along with over 800 million dry tonnes of agricultural residues (mostly straw) including over 113 million tonnes of rice husks (hulls) [1, 2].

Vietnam is a major rice grower and produces over 60 million tonnes of rice straw and husks every year. The bulk of this biomass is disposed of by burning, resulting in substantial emissions of black carbon, methane and the generation of tropospheric ozone leading to high levels of air pollution [3]. This has negative impacts on air quality and human health, reduces crop productivity and contributes to global warming [4, 5]. Decreasing emissions from burning agricultural waste, amongst other measures, should be adopted as a priority measure by the international community if we are to meet the proposed 2 °C target for limiting anthropogenic global temperature increases. Other potential disposal methods, such as incorporation into wet soil, are also responsible for increased methane emissions [6]. Hence, there is great interest in developing approaches to exploit the energy potential of such biomass, for example, through conversion to energy or to biofuels.

There have been a number of studies on the pretreatment, enzymatic saccharification and in some cases simultaneous saccharification and fermentation (SSF) of rice straw for the production of ethanol fuel. A range of pretreatments have been assessed, including steam explosion [7, 8], steam explosion and biological pretreatment [9], alkaline pulping [10], microwave alkali heating [3] organosolvent pretreatments [11] and fine milling [12]. There have been comparatively fewer studies on rice husk which have included investigations into microwave alkali heating [3], hydrogen peroxide treatments [13] and wet air oxidation [14]. Very few studies have used one of the most promising pretreatment technologies, steam explosion, for rice husks in relation to enzymatic saccharification with or without fermentation [15].

At a lab-scale, strong alkali and alkali-peroxide pretreatments typically perform well [15, 16]. However, commercial use of alkali at the levels used in the laboratory, usually at between 10 % [16] and 100 % [17] weight equivalent of the biomass, would require expensive waste water remediation to avoid pollution. Steam explosion, on the other hand, can be carried out without any added chemicals. Moreover, heat used for pretreatment can be recovered and used for downstream processes [18]. In addition, published studies on rice straw and husk that have been carried out in the laboratory are typically conducted at a very small scale <100–300 mL, and substrate concentrations generally <10 % (w/v). This potentially limits the relevance of these processes, when scaled up to industrial scales. Under commercial conditions, higher substrate concentrations would almost certainly be needed to produce suitable ethanol concentrations.

In the light of these discrepancies, and because studies on alkaline pretreatments suggest that rice husk is more difficult to saccharify effectively than rice straw [3, 15, 19], the aim of this research has been to carry out a carefully controlled comparative study of the effect of steam explosion pretreatment on enzymatic saccharification, and simultaneous saccharification and fermentation of rice straw and rice husk. We used a cultivar widely grown in North East Vietnam to evaluate saccharification of both substrates at small pilot scale. This has enabled us to identify key differences in processing capabilities between the two tissue types, and to demonstrate that at small pilot scale, steam exploded rice straw can be saccharified with minimal enzymes to produce industrially useful glucose concentrations.

## Methods

### Aim of the study and sourcing of biomass

The aim of this research has been to compare the effects of steam explosion pretreatment on enzymatic saccharification, and simultaneous saccharification and fermentation of rice straw and rice husk. Field grown rice (*Oryza sativa,* cv. KhangDan18) straw and husk was harvested at maturity in spring 2012 at Ba Vi, Hanoi, Vietnam. The biomass was fumigated and then air-dried under ambient conditions (approx. 34 °C, 84 % RH) at the Agricultural Genetics Institute (Hanoi, Vietnam). Before steam explosion pretreatment, the moisture content of the straw was determined to be 9.01 % and the husk 9.98 % (w/w).

### Steam explosion of rice straw and husk

Both rice straw and husk were steam exploded into hot water (70–80 °C) using a Cambi™ Steam Explosion facility with a 35 L reactor (Cambi AS, Asker, Norway). The rice straw was cut into lengths of 2–3 cm prior to loading the reactor. No size reduction was needed for the husk. The reactor was charged with 500 g of feedstock, sealed and heated with steam to the desired temperature (180–230 °C) at which it was retained for 10 min. After this time, the contents of the heating chamber were exploded into 3.5 L of hot water. The pretreated slurry was collected and fractionated into solid and liquid phases by centrifuging through a 100 µm nylon mesh. The insoluble residue was washed extensively to remove any water-soluble material. Both fractions were quantified, and samples stored at −40 °C. Portions of this material were freeze-dried for carbohydrate analysis.

### Carbohydrate composition of solids

Freeze-dried solids were acid hydrolysed (72 % (w/w) H_2_SO_4_, 3 h, RT followed by dilution to 98 g/L H_2_SO_4_, and heating for 2.5 h, 100 °C) to convert polymeric sugars into their monomeric constituents [20] . The hydrolysed samples were cooled on ice (>10 min) and then centrifuged (2500 rpm, 2 min). To a 1 mL aliquot from each hydrolysed sample 100 µL ribose internal standard (30 mg/mL) was added. Samples were neutralised with CaCO_3_ (2.5 mL, 2 mol/L). The precipitated salt was removed by centrifugation (3000 rpm, 10 min). Filter plates (AcroPrep™ 0.2 µm GHP Membrane 96 Well Filter Plates, VWR International Ltd, Lutterworth, UK) were used to filter portions of each sample (1 mL) prior to HPLC by centrifugation at 500 rpm for 10 min. Deep well collection plates were sealed with pierceable lids (Starlab (UK) Ltd, Milton Keynes, UK) and loaded directly onto Series 200 LC instrument (Perkin Elmer, Seer Green, UK) equipped with a refractive index detector and employing an Aminex HPX-87P carbohydrate analysis column (Bio-Rad Laboratories Ltd, Hemel Hempstead, UK) with matching guard columns.

### Small scale saccharification

Small scale saccharification was carried out in 25 mL universal vials (Sterilin, Newport, Gwent, UK), hydrolysing 1 g DM equivalent of wet pretreated solid, made to 5 % substrate concentration with sodium acetate/acetic acid buffer (8.2 g/L, pH 5.0). The buffer contained 0.01 % (w/v) thiomersal to prevent microbial contamination. Hydrolyses were conducted for 96 h at 50 °C, under continuous agitation in a Thermoshake Incubating Orbital Shaker (Gerhardt, Königswinter, Germany) after adding an appropriate amount of cellulase. The two commercially available enzyme preparations used in this study were Cellic^®^ CTec2 assayed following Ghose [21] and Cellic^®^ HTec2 (Novozymes, Bagsvaerd, Denmark). Hydrolysis reactions were terminated by heating the hydrolysate in sealed tubes (100 °C, 10 min) after which the samples were cooled, centrifuged (2000 rpm, 2 min 25 °C) and the supernatants recovered for analysis.

### Small scale simultaneous saccharification and fermentations

Small scale simultaneous saccharification and fermentation (SSF) was conducted in 30 mL wide-necked glass vials containing 1 g (DM equivalent) of wet pretreated solid, made up to 17.9 mL with yeast nitrogen base (Formedium, Hunstanton, UK) at pH 5.0. The bottles were then autoclaved (121 °C, 15 min) to ensure sterility. The bottles were cooled to 25 °C, and 2 mL of yeast grown in Difco YM media (Fisher Scientific UK Ltd, Loughborough, UK), was added along with 100 µL Cellic^®^ CTec2 (Novozymes, Bagsvaerd, Denmark), 20 FPU/g substrate. The yeast inoculum used was a *Saccharomyces cerevisiae* strain—NCYC 2826—chosen from the National Collection of Yeast Cultures (UK), selected on the basis of its high ethanol tolerance (15–20 % v/v). The inoculum had a viable cell count of 9.87 × 10^7^ cells/mL. Bottles were incubated under continuous agitation (120 h, 25 °C) after which, a measured sample was boiled in gas tight screw cap tubes (Starlab Ltd, Milton Keynes, UK), centrifuged (13,000 rpm, 5 min) and supernatant filtered through AcroPrep™ 0.2 µm GHP Membrane 96 Well Filter Plates (VWR International Ltd, Lutterworth, UK) into a 96 deep well collection plate before analysis.

### Five litre pilot scale hydrolyses in high torque bioreactor

Pilot scale hydrolyses (5 L) were conducted in a bespoke high torque bioreactor [22]. Digests were conducted at 20 % (w/v) substrate concentration, using 1 kg DW equivalent of steam exploded rice straw/husk suspended in sodium acetate/acetic acid buffer (4.1 g/L, pH 5.0). The buffer and substrate were initially heated to >85 °C for 10 min to minimise the possibility microbial contamination. The mixture was then cooled to 50 °C and an appropriate amount of Cellic^®^ CTec2 was added—the optimum was either 6.49 or 10 FPU/g DM for straw and husk, respectively. The hydrolysate was agitated at 39 rpm for 4 days, taking 100 mL samples of the digest every 24 h. Each sample was heated in a sealed tube to (100 °C, 10 min), centrifuged (2000 rpm, 2 min) and the monomeric sugar composition determined.

### Quantification of hydrolysis and fermentation products

The concentration of reducing sugars released from the substrates was quantified using a scaled dinitrosalicylic acid (DNS) method [23]. Glucose concentrations were quantified using a glucose-specific kit (GOPOD, Megazyme International Ireland, Bray, Ireland). Substrate and enzyme controls were included wherever necessary.

Terminated fermentations, after centrifugation and filtration through a 0.2 μm filter, were loaded directly onto a Series 200 LC instrument (Perkin Elmer, Seer Green, UK) equipped with a refractive index detector. The analyses were carried out using an Aminex HPX-87P carbohydrate analysis column (Bio-Rad Laboratories Ltd, Hemel Hempstead, UK) with matching guard columns operating at 65 °C with ultrapure water as mobile phase at a flow rate of 0.6 mL/min as described [22].

### Quantification of fermentation inhibitors

Pretreatment-derived supernatants were recentrifuged (2465×*g*) and 200 μL of the supernatant syringe filtered into vials (0.2 µm, Whatman International Ltd, Maidstone, UK). The concentration of the fermentation inhibitors 2-furfuraldehyde (2-FA), 5-Hydroxymethylfurfural (5-HMF) and the organic acids (formic and acetic acid) were analysed by HPLC using a Flexar LC instrument (PerkinElmer, Seer Green, Bucks., UK) equipped with refractive index and photo diode array detectors (reading at 210 nm wavelength) in series. The analyses were carried out using an Aminex HPX-87H carbohydrate analysis column (Bio-Rad Laboratories Ltd, Hemel Hempstead, UK) operating at 65 °C with 0.005 mol/L H_2_SO_4_ (Sigma-Aldrich) as mobile phase at a flow rate of 0.6 mL/min.

### Statistical analysis

Unless otherwise stated, all analyses were performed in triplicate and presented as means and standard deviations. Curve fitting was conducted in Genstat v.18 (VSN International).

## Results and discussion

### Effect of pretreatment on the recovery and chemical composition of rice straw and husk

**Fig. 1:**
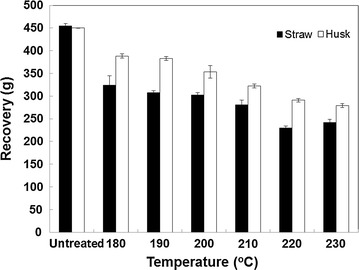
Yield of rice straw and rice husk dry matter after steam explosion. Rice straw and rice husk were steam exploded into hot water after a residence time of 10 min at 180–230 °C. The residues were washed and aliquots freeze-dried

Rice straw and husk samples were pretreated by steam explosion at temperatures of between 180 and 230 °C after which they were exploded into hot water. At the lower intensities (180–190 °C for 10 min), the steam exploded materials retained considerable structure, and were slightly darkened in colour compared with the control. At higher severities, the biomass became much darker in colour and lost most of its structure, with an increase in fine particulate matter. The yields of insoluble dry matter recovered are shown in Fig. 1. The decrease in insoluble dry matter at higher severities was due to a combination of solubilisation of cell wall components (predominantly hemicelluloses, lignin and salts), hydrolysis, breakdown and some volatilisation of wall components. At the highest severities, additional losses of fine particulate matter through the recovery mesh and the loss of some insoluble solids from the cyclone in the steam exploder were more likely.

**Fig. 2:**
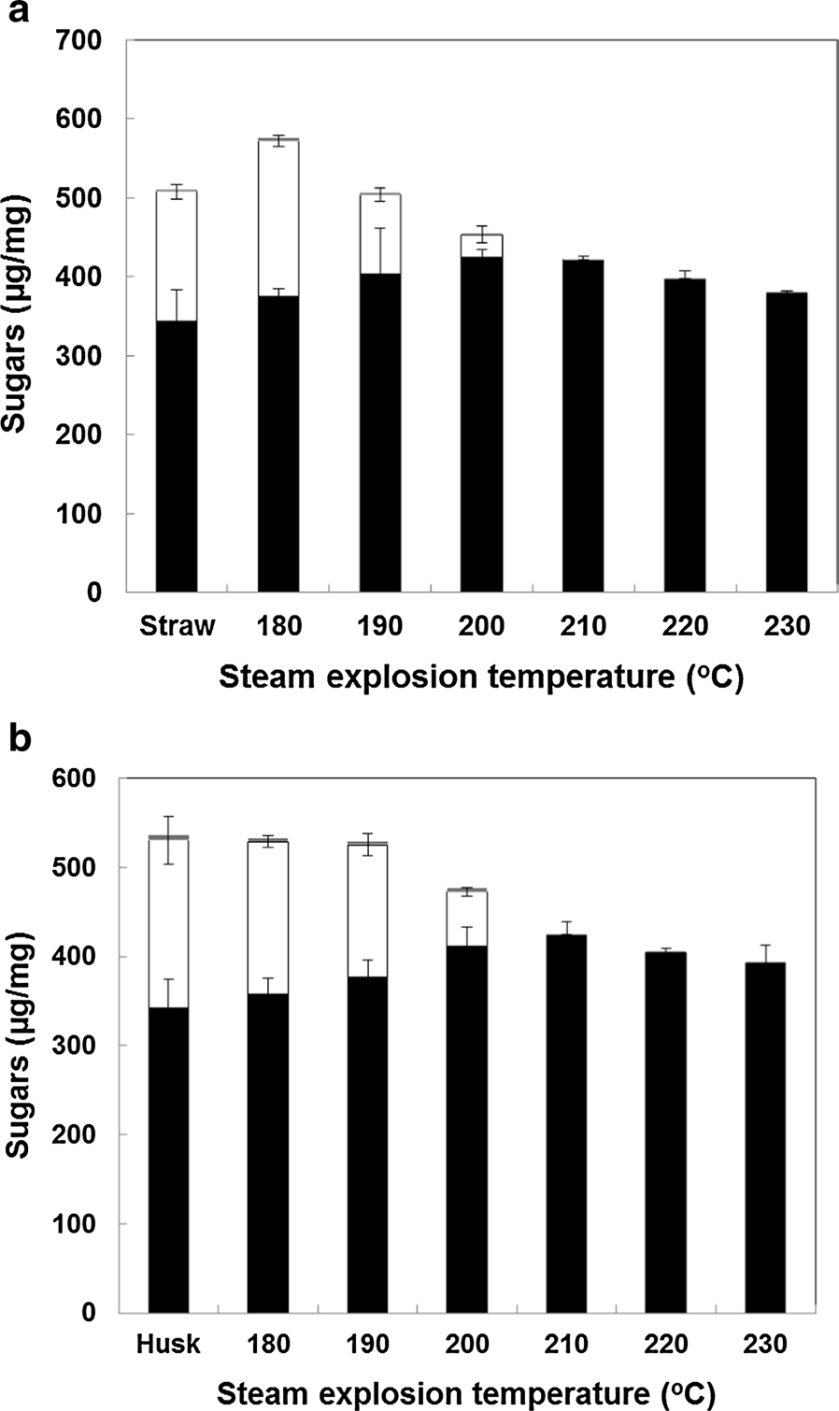
Composition (µg/mg dry matter) of main sugars (glucose, *black bars*; xylose, *white bars*) in (**a**) steam exploded rice straw and (**b**) rice husk. Samples were hydrolysed in hot sulphuric acid, aliquots neutralised and then sugars quantified by HPLC

The main cell wall sugars present in the raw and pretreated straw and husk residues are shown in Fig. 2. The levels of cellulosic glucose and hemicellulosic xylose are similar to those reported previously for rice straw [3, 24, 25] and hulls [3, 13]. The steam explosion pretreatment and washing reduced the levels of insoluble hemicellulosic xylose as severity increased. Xylose was completely removed by steam explosion at temperatures at or above 210 °C. This is consistent with previous observations [7] and is similar to the effects of steam explosion on wheat straw [26]. Other neutral sugars such as arabinose and galactose were present at very low levels and are not shown. The loss of the hemicellulosic components and probably some of the lignin resulted in an increase in the proportions of (cellulosic) glucose. However, this peaked under treatment conditions of 200 °C for 10 min for rice straw, and 210 °C for 10 min for rice hull after which it declined, probably due to breakdown of some of the cellulose. The high-severity related loss of sugars also resulted in an increase in the levels of breakdown products (including fermentation inhibitors) recovered in the aqueous phase. These were measured by HPLC and the results highlight the production of formic acid, acetic acid, 5-HMF and 2-FA (Table 1). Interestingly, the rice straw and hull differed significantly in their production of breakdown products. At the low severities, more 5-HMF and 2-FA were released from the straw as compared with the hull. However, at the higher severities, this was reversed. The implications of this are discussed below.

**Table 1:** Quantity of organic acid held in the pretreatment liquor, expressed as g per kg of original straw

Steam explosion temperature (°C^a^)		Formic	Acetic	5-HMF	2-FA
180					
Straw		17.30 ± 0.34	11.76 ± 0.25	1.34 ± 0.00	1.80 ± 0.14
Husk		3.57 ± 0.05	2.52 ± 0.07	0.51 ± 0.05	0.41 ± 0.00
190					
Straw		16.56 ± 0.05	10.21 ± 0.06	1.21 ± 0.04	2.07 ± 0.07
Husk		8.12 ± 0.16	8.06 ± 0.06	0.88 ± 0.02	2.19 ± 0.00
200					
Straw		16.60 ± 0.94	9.37 ± 0.66	1.50 ± 0.08	1.43 ± 0.08
Husk		6.96 ± 0.41	8.15 ± 0.81	1.18 ± 0.05	3.21 ± 0.51
210					
Straw		15.05 ± 0.30	9.78 ± 0.11	1.30 ± 0.04	1.19 ± 0.04
Husk		6.12 ± 2.40	8.24 ± 2.57	1.58 ± 0.33	2.69 ± 0.78
220					
Straw		13.60 ± 0.69	8.26 ± 0.41	0.99 ± 0.08	0.75 ± 0.03
Husk		6.87 ± 0.03	11.07 ± 0.04	2.58 ± 0.00	2.56 ± 0.02
230					
Straw		1.81 ± 0.09	1.77 ± 0.05	0.35 ± 0.00	0.37 ± 0.02
Husk		N.D.	N.D.	N.D.	N.D.

^a^Residence time in all cases was 10 min

### Enzymatic saccharification—impact of pretreatment

**Fig. 3:**
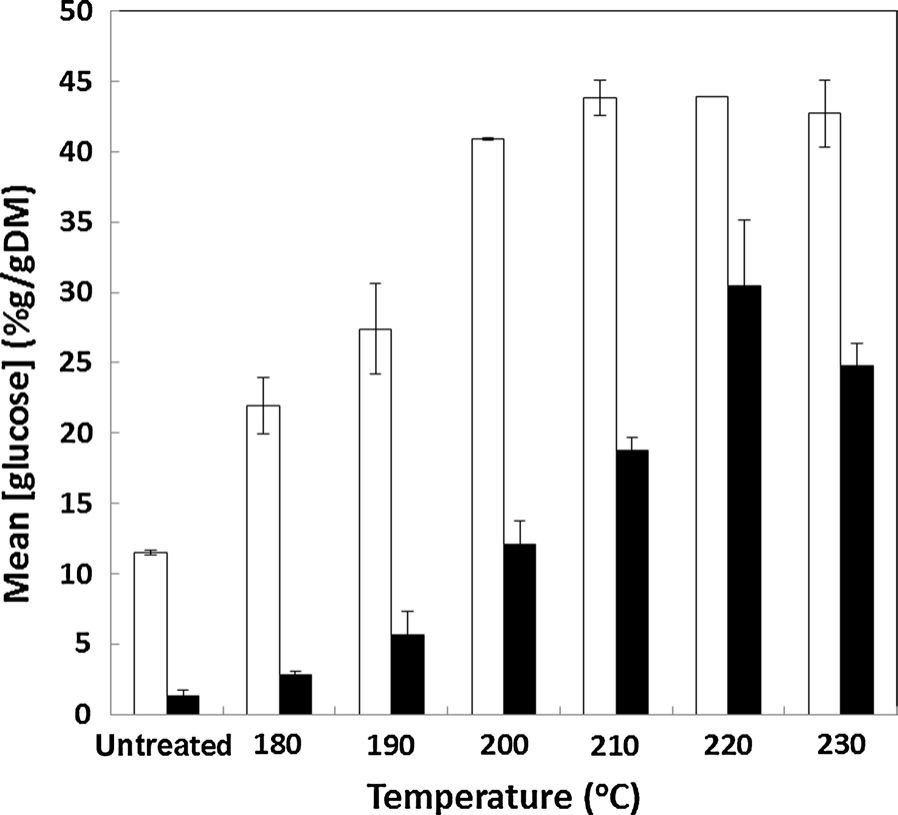
Impact of pretreatment severity on saccharification (glucose release) of rice straw (*white bars*) and rice husk (*black bars*); n = 2. Samples were digested whilst being agitated at a substrate concentration of 5 % (w/v) at 50 °C for 96 h in cellulase (Cellic^®^ CTec2; circa 25 FPU/g cellulose)

Pretreated, insoluble straw and husk residues were subjected to an extended digestion in cellulase Cellic^®^ CTec2 at a loading of 10 FPU/g DM (circa 25 FPU/g cellulose) for 96 h at 50 °C whilst being continually agitated. The glucose released was measured using the specific GOPOD assay (see “Methods” section) and the results are shown in Fig. 3. At all severities, the glucose released from rice straw was of a greater yield compared with that from rice husk. This does not, however, reflect variation in the levels of cellulosic glucose present in the substrates (Fig. 2). The highest yields of glucose from straw were achieved after severities at or greater than 210 °C for 10 min (Fig. 3). Higher severities had no additional impact, and indeed suggested a (nonsignificant) reduction in yield, consistent with the slightly lower levels of substrate glucose shown in Fig. 2a. The results indicate that virtually all of the measurable glucose present in the pretreated straw was released under such conditions.

**Fig. 4:**
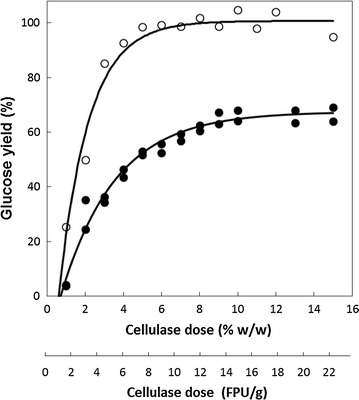
Optimisation of enzyme loading for saccharification. Yields of glucose achieved when hydrolysing rice straw (*circle*) and husk (*filled circle*) steam exploded at optimum conditions (10 min at 210 and 220 °C, respectively). Glucose yields are expressed as a percentage of the maximum theoretical yield. Samples were digested whilst being agitated at a substrate concentration of 5 % (w/v) at 50 °C for 96 h in cellulase (Cellic^®^ CTec2; between 0 and circa 22 FPU/g cellulose)

In comparison, the saccharification of rice husk was much less efficient. The maximum enzymatic release of glucose required a higher pretreatment severity (220 °C for 10 min) and the extent of saccharification was even then only about 75 % that of the straw. Increasing the severity to 230 °C for 10 min resulted in a significant reduction in glucose yield. In comparison, Pineros-Castro et al. [15] studied the effect of acid-catalysed steam explosion of rice husk on enzymatic saccharification. They impregnated the husks with 0.5 % (w/v) sulphuric acid and steam exploded at 190 °C for between 10 and 25 min. After saccharification with high levels of cellulase (Celluclast^®^ at 15 FPU/g), they achieved a maximum glucose yield of only 15 % (w/w), which from the chemical composition of the initial starting material of 35 % (w/w) was less than half of the theoretical yield and much less than that achieved in this study. Cabrera et al. [19] compared the impact of alkaline and alkaline peroxide pretreatments at lower temperatures to enhance enzymatic hydrolysis of rice hulls and straw. They also employed a high enzyme loading (circa 20 FPU/g cellulose) for 72 h on pretreated and insoluble biomass and found that whilst they were able to achieve over 90 % hydrolysis of reducing sugars from rice straw, rice hulls gave a yield of 77.5 % which is similar to this study but necessitates the use of polluting alkali. Singh et al. [3] evaluated enzymatic saccharification of rice straw and hull after microwave alkali pretreatment. In that study, they produced enzymes in situ by incubating the pretreated residues with *Aspergillus heteromorphus* and monitored digestion and the release of reducing sugars. In pretreated rice straw, up to 35 mg/g sugars were released whilst in hull, this was limited to about 22 mg/g. Hence, across all pretreatment conditions studied including this study on steam explosion, the cellulose from rice straw is much more readily hydrolysable than the cellulose from rice husk.

### Enzymatic saccharification—optimisation of enzyme loadings

On the basis of the data above, material pretreated at 210 and 220 °C was employed for the optimisation of enzyme loading. Pretreated material (5 % w/v) was digested with cellulase (Cellic^®^ CTec2) for 96 h at 50 °C using a range of loadings after which the release of glucose was determined (Fig. 4). For pretreated rice straw (210 °C, 10 min), the minimum loading of cellulase required to obtain maximum glucose release was 7 FPU/g DM (circa 18 FPU/g cellulose). In contrast, the pretreated rice husk required a higher loading in excess of 10 FPU/g DM (circa 25 FPU/g cellulose). Consistent with the results in Fig. 3, the maximum yield of glucose from rice husk was also much less than that of straw highlighting its relatively lower suitability as a substrate.

**Fig. 5:**
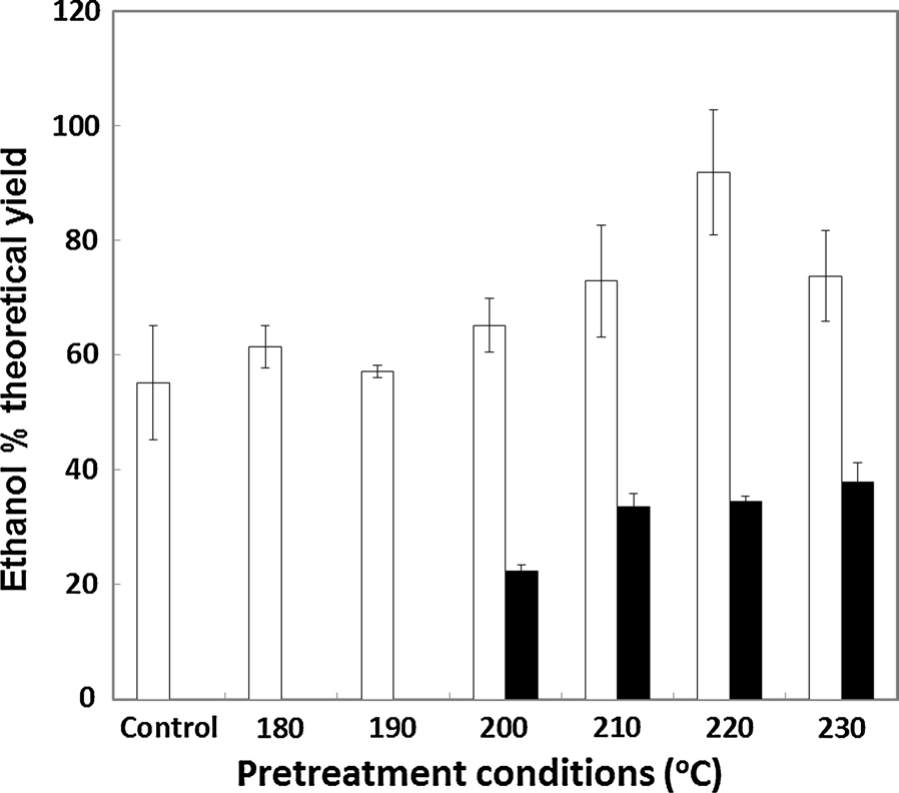
Simultaneous saccharification and fermentation (SSF) of rice straw and rice husk for 96 h after steam explosion pretreatment at different severities. SSF was carried out with 5 % (w/v) substrate, cellulase (Cellic^®^ CTec2; circa 20 FPU/g dry matter) and yeast (NCYC 2826) at 25 °C and pH 5

Novozymes Cellic^®^ CTec2 can be augmented by a cocktail of accessory enzymes (Cellic^®^ HTec2) which contains hemicellulose-degrading enzymes such as xylanases [27]. The addition of Cellic^®^ HTec2 on the efficiency of saccharification of pretreated rice straw and husk was also evaluated by substituting Cellic^®^ CTec2 with increasing levels of Cellic^®^ HTec2. However, Cellic^®^ HTec2 addition had no significant impact on the efficacy of Cellic^®^ CTec2 on saccharification (Additional file 1: Figure S1 a, b). This is consistent with the prior removal of any sterically inhibiting hemicelluloses by the steam explosion pretreatment and could have an economically beneficial impact on the overall cost of enzymes used in the process.

### Simultaneous saccharification and fermentation

The potential for conversion of the cellulosic components of pretreated rice straw and husk to ethanol was also evaluated at small scale, under simultaneous saccharification and fermentation (SSF) conditions. Weighed aliquots of pretreated biomass (1 g DM, wet state) were made up to 5 % (w/v) in yeast nitrogen base, autoclaved, cooled to 25 °C, and inoculated with a high ethanol tolerance yeast strain and excess Cellic^®^ CTec2 to 20 FPU/g DM. SSF was carried out at 25 °C for 120 h after which the levels of ethanol in the supernatant were measured by HPLC. The results are shown in Fig. 5. The yield of ethanol from rice straw appears to be optimal in the material pretreated at 220 °C, perhaps slightly higher than observed from saccharification alone (210 °C; Fig. 3).Thus, pretreatment in the range of 210–220 °C enables quantitative conversion of cellulosic glucose to ethanol. This was not the case for rice husk which produced particularly low ethanol yields. Low severity pretreatment during the autoclave sterilisation process may explain why the untreated rice straw yielded higher ethanol yields than might be expected when compared with the saccharification data.

### Enzymatic saccharification—increasing the concentration of glucose released

**Fig. 6:**
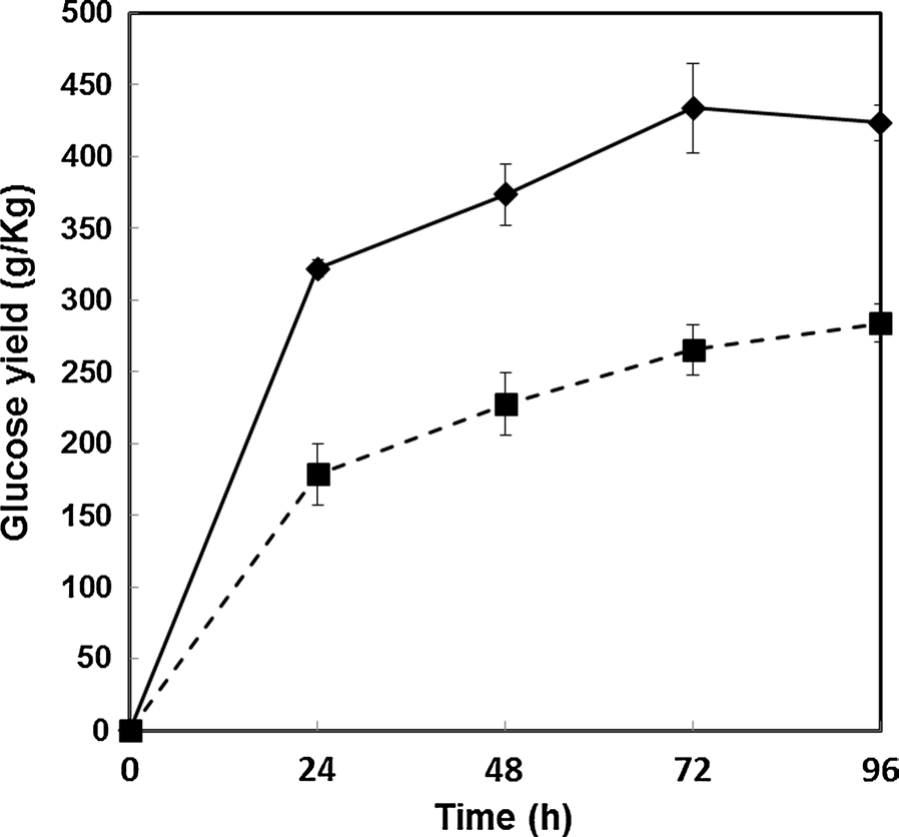
Time course of glucose yields (g/kg) from pretreated straw and husk at small pilot scale (the glucose reached a concentration of 9 % w/v) n = 5. Saccharification was conducted at 20 % (w/v) substrate concentration, using 1 kg DW equivalent of steam exploded rice straw/husk (pH 5.0). After a pasteurisation at 90 °C, the mixture was cooled to 50 °C and Cellic^®^ CTec2 was added. The hydrolysate was agitated at 39 rpm for 4 days, sampling every 24 h

The bulk of academic studies on saccharification of lignocellulose predominantly involve low substrate concentrations to enable good mixing in suspension. To provide a comparison of straw and husk saccharification under more industrially relevant conditions, a small pilot scale study was performed at 10 L using a scalable high torque bioreactor developed previously [22, 28]. This enabled digests to be carried out at a high solids loadings of 20 % (w/v) which would not have mixed effectively on a normal bench shaker incubator or stirrer due to the high viscosity. Bulk quantities of straw and husk were pretreated at the optimal conditions specified, and optimised Cellic^®^ CTec2 enzyme loadings were used for rice straw (6.49 FPU/g DM) and husk (10 FPU/g DM). Samples were taken regularly over a 96 h period and analysed for the release of monomeric glucose. The results (Fig. 6) clearly show that at high substrate concentrations, the cellulose in pretreated rice straw can be quantitatively saccharified to glucose with a yield of approximately 100 % within 72 h. This gave a final glucose concentration of 9 % (w/v). Pretreated rice husk, reflecting the smaller scale evaluations, was not as effectively saccharified, reaching a plateau at much lower glucose concentrations after >96 h of incubation.

The increasing interest in processes that involve high solids loadings to achieve higher product concentrations relates to the lower capital costs due to the requirements for smaller volume or fewer reactors, lower energy consumption for the reactions, and a reduction in water waste disposal costs [29]. Liu et al. [30], for example, reported laboratory (small scale 250 mL) studies on batch-fed saccharification of sugarcane bagasse, and achieved 135 g/L glucose (13 % w/v) using cellulase loadings of 8.5 FPU/g substrate. Their pretreatment required high levels of hot alkali (equivalent to 200 kg NaOH per tonne bagasse). An earlier report by Yang et al. [31] likewise achieved 175 g/L glucose from batch addition of pretreated corn stover in the presence of cellulase loadings of 20 FPU/g substrate. Their pretreatment involved extensive peroxide and alkali treatments. The advantage of such pretreatments is that they extract much of the lignin, and produce substrates containing over 60 % (w/w) cellulose. The outweighing disadvantage, however, is that industrial exploitation would lead to a high level of salt pollution from the alkali used. In this study, we have used steam explosion, without the addition of other chemicals as a pretreatment, and a high torque bioreactor to handle a high solids loading with low enzyme levels (6 FPU/g substrate for rice straw, 10 FPU/g substrate for rice husk) to produce a high glucose yield, with potential for increasing it further through batch addition [22]. We have also definitively demonstrated that rice husk is much more difficult to saccharify than straw, directly impacting ethanol yield during simultaneous saccharification and fermentation.

## Conclusions

The aim of this study has been to systematically compare the effects of uncatalysed steam explosion on the enzymatic saccharification and simultaneous saccharification and fermentation of rice straw and husk produced from the single variety, widely grown in Vietnam (*O. sativa*, cv. KhangDan18). In spite of having similar carbohydrate compositions, rice husk is much less amenable to saccharification even at optimal pretreatments when compared with rice straw. The limits to saccharification posed by rice husk cannot be solved by increases in cellulase or hemicellulase abundance. This result, combined with those from other studies using various pretreatments, suggest that the utilisation of rice husk as a biorefinery feedstock would be difficult to achieve in its native form. However, rice husk is produced in vast tonnages and is thus a prime target for exploitation. Therefore, breeding strategies aimed to reduce the recalcitrance of this abundant material, and further developments in pretreatment technologies such as AFEX [32, 33] would be appropriate.

## Additional file

10.1186/s13068-016-0599-6 % yield of glucose released from (a) pretreated straw and (b) pretreated husk during enzyme hydrolysis over 96 h as modulated by increasing concentrations of Cellic^®^ HTec2 [0 %; 5 %; 10 %; 15 %: Cellic^®^ HTec 2 concentration (% g/g Cellic^®^ CTec2)]. Samples were digested whilst being agitated at a substrate concentration of 5 % (w/v) at 50 °C for 96 h in cellulase (Cellic^®^ CTec2; between 0 and circa 22 FPU/g cellulose).

## Abbreviations

DM: dry matter
FPU: filter paper units
SSF: simultaneous saccharification and fermentation
