# Supplementary material for: Comparison of saccharification and fermentation of steam exploded rice straw and rice husk
Source: Biotechnol Biofuels. 2016 Sep 5;9(1):193. doi: 10.1186/s13068-016-0599-6 (PMC5011935; doi:10.1186/s13068-016-0599-6)
Supplement: Supplementary file 1 — 10.1186/s13068-016-0599-6 % yield of glucose released from (a) pretreated straw and (b) pretreated husk during enzyme hydrolysis over 96 h as modulated by increasing concentrations of Cellic® HTec2 [0 %; 5 %; 10 %; 15 %: Cellic® HTec 2 concentration (% g/g Cellic® CTec2)]. Samples were digested whilst being agitated at a substrate concentration of 5 % (w/v) at 50 °C for 96 h in cellulase (Cellic® CTec2; between 0 and circa 22 FPU/g cellulose). [file 13068_2016_599_MOESM1_ESM.docx]

***Supplementary Figure. S1.*** *% yield of glucose released from (a) pretreated straw and (b)pretreated husk during enzyme hydrolysis over 96 h as modulated by increasing concentrations of HTec2. (0%; 5%; 10%, 15%: Htec 2 concentration (% g/g CTec2))*

**
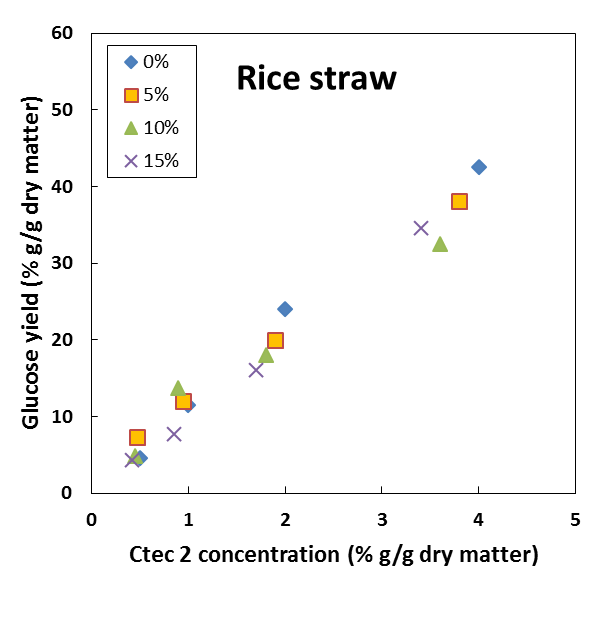
(a)**

**
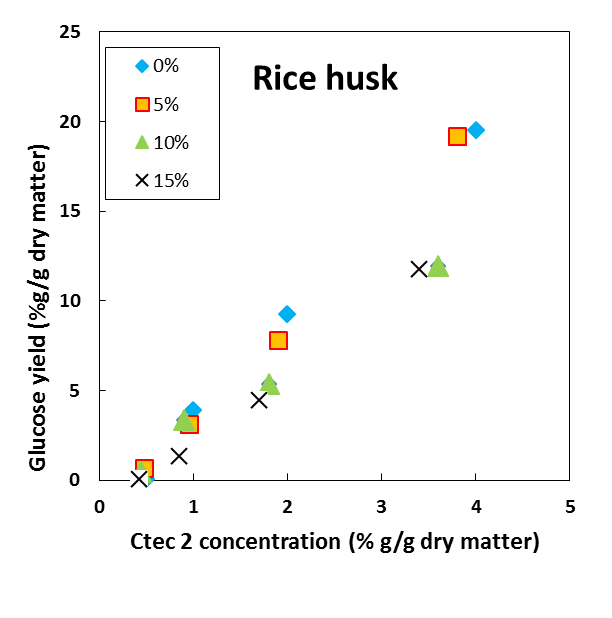
(b)**
